# Effects of intermittent hypoxia and hypoxia–hyperoxia exposure on recovery from delayed onset muscle soreness in physically active men: protocol for a randomized controlled trial

**DOI:** 10.3389/fspor.2026.1768915

**Published:** 2026-03-25

**Authors:** Sebastian Philippe Hansen Quiblier, Guillermo García-Pérez-de-Sevilla, Catalina Santiago-Dorrego, Tamara Iturriaga Ramírez, Rebeca Benítez-Valero, Carlos Revuelta Parra, Alejandro Fernández Carrasco, Diego Domínguez-Balmaseda

**Affiliations:** 1 Department of Real Madrid Graduate School, Faculty of Medicine, Health and Sports, Universidad Europea de Madrid, Madrid, Spain; 2 Department of Physiotherapy, Faculty of Medicine, Health and Sports, Universidad Europea de Madrid, Madrid, Spain; 3 Department of Sports Sciences, Faculty of Medicine, Health and Sports, Universidad Europea de Madrid, Madrid, Spain

**Keywords:** athletes, delayed onset muscle soreness, hypoxia–hyperoxia, intermittent hypoxia, recovery

## Abstract

**Introduction:**

The objective of this study is to evaluate whether intermittent hypoxia can enhance physical recovery following the induction of delayed onset muscle soreness (DOMS).

**Methods and analysis:**

A randomized controlled trial will be conducted with male participants aged 18–35 years who engage in at least two organized training sessions per week. After a DOMS induction protocol targeting the hamstrings, participants will be randomized to receive either intermittent hypoxia, hypoxia–hyperoxia, or placebo for five consecutive days. During this period, several outcome variables will be assessed, including: cognitive function—attention and executive functions (D2 Test and Trail Making Test A and B); stress (DASS-21 questionnaire); physical performance—VO_2_max, countermovement jump (CMJ, My Jump App), 30-m sprint speed (MySprint App), one-repetition maximum in half-squat (Vitruve linear encoder), isometric hamstring strength (ActivForce2 dynamometer), muscle soreness (VAS scale), heart rate variability, muscle oxygen saturation, hip flexion range of motion with extended knee (Goniometer Pro App); and blood inflammatory markers: HIF-1α, PGC-1α, Klotho protein and lactate. To analyze the effects of the intervention, a repeated-measures ANOVA will be performed to assess Group × Time interactions.

**Ethics and disseminations:**

The study was reviewed and approved by the Medical Ethics Committee of Hospital Clínico San Carlos, Madrid, Spain (internal code 25/446-E) prior to data collection. If effective, intermittent hypoxia and hypoxia-hyperoxia could be implemented as non-pharmacological recovery strategies for athletes and physically active populations, offering a novel alternative to current methods with inconsistent results. The findings may guide future research on hypoxic conditioning, inform clinical practices for rehabilitation and performance recovery, and potentially shape policy regarding safe, evidence-based applications of hypoxia in sports medicine.

**Clinical Trial Registration:**

https://www.anzctr.org.au/, registration code ACTRN12625001404415.

## 1 Introduction

Delayed onset muscle soreness (DOMS) triggers increased neural activity driven by inflammatory mediators, negatively impacting performance (1–3). Although several approaches have been investigated to reduce DOMS, their effectiveness varies, yielding inconsistent results across different demographic groups and exercise regimens (4).

Intermittent hypoxia (IH) is an emerging strategy that alternates between exposure to hypoxic environments (low oxygen availability) and normoxic environments (normal oxygen availability, similar to altitudes below 1,800 meters) (5). Originally developed to mimic the benefits of high-altitude training, IH has demonstrated several physiological advantages, including improved mitochondrial function, reduced inflammation, enhanced oxygen transport, and increased capillary formation—all of which are critical for muscle recovery (6). Moreover, the activation of hypoxia-inducible factors (HIFs) in response to IH has been linked to favorable adaptations that promote recovery and enhance performance (7). Beyond muscle regeneration, IH also influences oxidative stress and inflammation, two key contributors to DOMS (8). Research suggests that controlled hypoxic exposure can strengthen the body's antioxidant defenses and reduce the production of proinflammatory cytokines (9). This implies that IH could potentially mitigate the pain, stiffness, and inflammation associated with DOMS, although no published studies have yet addressed this topic.

Another promising approach is hypoxia–hyperoxia (HH), which alternates between hypoxic (low oxygen availability) and hyperoxic (high oxygen availability) conditions (10). The rapid transition between hypoxia and hyperoxia is believed to stimulate angiogenesis, mitochondrial biogenesis, and metabolic efficiency, all of which facilitate adaptation and muscle recovery (11). The additional oxygen surge during the hyperoxic phase may also enhance tissue oxygenation, support the clearance of metabolic waste, and reduce muscle fatigue (12). A key element in this process is the activation of hypoxia-inducible factor 1-alpha (HIF-1α), the central regulator of cellular responses to low oxygen availability (13). HIF-1α promotes the expression of peroxisome proliferator-activated receptor gamma coactivator 1-alpha (PGC-1α), a critical regulator of mitochondrial biogenesis (6, 9). Moreover, DOMS is also associated with global fatigue and altered sensorimotor and perceptual states, which may transiently affect central processes such as attention (14). Emerging evidence indicates that hypoxic-based interventions, including intermittent HH, modulates neural, autonomic, and inflammatory pathways, and could simultaneously affect both physical recovery from DOMS and cognitive processes impaired during post-exercise fatigue (15). Accelerating recovery from DOMS may therefore contribute not only to restored sports performance but also to the recovery of attentional and cognitive functions, which are recognized as relevant components within the multifactorial determinants of athletic performance and injury risk (16).

The novelty of this research lies in exploring IH and HH exposure as comprehensive methods for muscle recovery, as they may influence not only muscle repair but also cognitive recovery and mental performance, which are often impaired by DOMS (17). Beyond localized muscle soreness, DOMS induces systemic and peripheral inflammatory responses and sustained nociceptive signaling that engage central somatosensory and motor processing networks, potentially influencing cognitive and psychomotor performance during recovery from intense exercise (18). The purpose of this study is to determine whether HH and IH can be effective techniques for DOMS recovery. Our hypothesis is that both HH and IH will accelerate recovery from DOMS. Additionally, given the alternating hyperoxic stimulus and the potentially amplified cellular signaling associated with hypoxia–hyperoxia protocols, we hypothesize that hypoxia–hyperoxia may induce greater improvements in recovery-related outcomes than intermittent hypoxia alone (17).

## 2 Methods

### 2.1 Study setting

This study is a prospective, longitudinal, randomized controlled trial conducted at Universidad Europea de Madrid, utilizing the university's physiology and sports performance laboratories. The trial will be carried out in accordance with the CONSORT guidelines (19).

The study will adhere to the ethical principles outlined in the Declaration of Helsinki. Prior to participation, all subjects will provide written informed consent, confirming that they understand the study procedures, potential risks, and their right to withdraw at any time. The study protocol was reviewed and approved by the Medical Ethics Committee of Hospital Clínico San Carlos, Madrid, Spain (internal code 25/446-E).

### 2.2 Participants and eligibility criteria

Participants in this study will be men aged 18–35 years who engage in at least two organized training sessions per week. Recruitment will take place through university teams at Universidad Europea de Madrid (Spain), including sports clubs and training groups.

Participation will be entirely voluntary, and prior to enrollment, individuals will be required to sign informed consent forms after receiving a comprehensive explanation of the study objectives, procedures, and potential risks.

Only male participants will be included in order to reduce physiological variability associated with sex-specific hormonal fluctuations that may influence inflammatory responses, muscle damage markers, and autonomic regulation following eccentric exercise (20). In addition, hypoxic exposure can induce cardiovascular and ventilatory adaptations that may be modulated by hormonal status, potentially introducing additional variability in mixed-sex samples (7, 20).

Exclusion criteria will include: prior exposure to hypoxia training (to avoid pre-existing adaptations to hypoxia); a history of respiratory conditions contraindicating hypoxia exposure, such as asthma or chronic obstructive pulmonary disease; use of anti-inflammatory medication or other treatments that could interfere with recovery (e.g., cold water inmersion, massage therapy); the presence of musculoskeletal, neurological, or cardiovascular disorders that could impair recovery; and a diagnosis of schizophrenia—not due to the intervention itself, but because the use of a mask could potentially trigger an episode.

### 2.3 Blinding and randomization

After recruitment, participants will complete the International Physical Activity Questionnaire (IPAQ). The sample will be matched by age and prior physical activity levels before randomization. Randomization will then be performed using a computer-generated random sequence created with Microsoft Excel, applying a 1:1:1 allocation ratio to assign participants to the intermittent hypoxia, hypoxia–hyperoxia, or placebo groups (19).

Participants will be allocated to coded groups (e.g., Group A, B, or C), without any indication of the intervention received. To ensure allocation concealment, the randomization sequence will be generated and managed by an investigator not involved in participant recruitment, data collection, or outcome assessment. Group assignments will be stored in a password-protected file and revealed only to the technician responsible for operating the hypoxic system, who will not participate in any assessments or data analysis (19).

To further reduce bias, the study will employ a triple-blind design: participants will not know their group allocation, evaluators will be unaware of the intervention administered to each subject, and data analysts will work exclusively with coded datasets without access to group identities.

#### 2.3.1 Participant blinding

All participants will use the same gas administration system (MITOVIT Hypoxic Training System, MITOVIT, Germany) with identical masks and external configurations, regardless of group allocation (IH, HH, or placebo). The device interface will remain concealed during the intervention, and perceptual differences between gas mixtures (FiO_2_ ≈ 12%, 21%, or 30%–35%) are minimal, thereby substantially reducing the likelihood that participants will consciously identify their experimental condition.

#### 2.3.2 Evaluator blinding

Personnel responsible for data collection—including physical, cognitive, psychological, and biochemical assessments—will be blinded to participants’ group assignments. To avoid inadvertent unblinding (e.g., when a protocol involves cycling exercise), the staff operating and supervising the MITOVIT Hypoxic Training System will not overlap with those conducting outcome assessments.

#### 2.3.3 Data analyst blinding

The dataset will be delivered to the statistician in coded format, using neutral identifiers (e.g., Group A, B, C). No information that could reveal the intervention type will be included. The correspondence between group codes and interventions will be known only to an external technician not involved in data collection or statistical analysis.

### 2.4 Sample size

The primary outcome of this trial is hamstring isometric strength. The required sample size was determined *a priori* using G*Power 3.1 considering a repeated measures of variance (ANOVA) design. In the absence of prior data to inform the expected magnitude of change, a conventional medium effect size (*f* = 0.25), as defined by Cohen, was assumed. With a significance level of *α* = 0.05, a statistical power of 0.80 (*β* = 0.20), and six time points, the analysis indicated that 36 participants would be required to detect statistically significant differences between groups. To account for potential dropouts and non-compliance, an additional 20% contingency was applied, increasing the total sample size to 45 participants (15 per group).

### 2.5 Interventions and protocol

**Figure 1:**
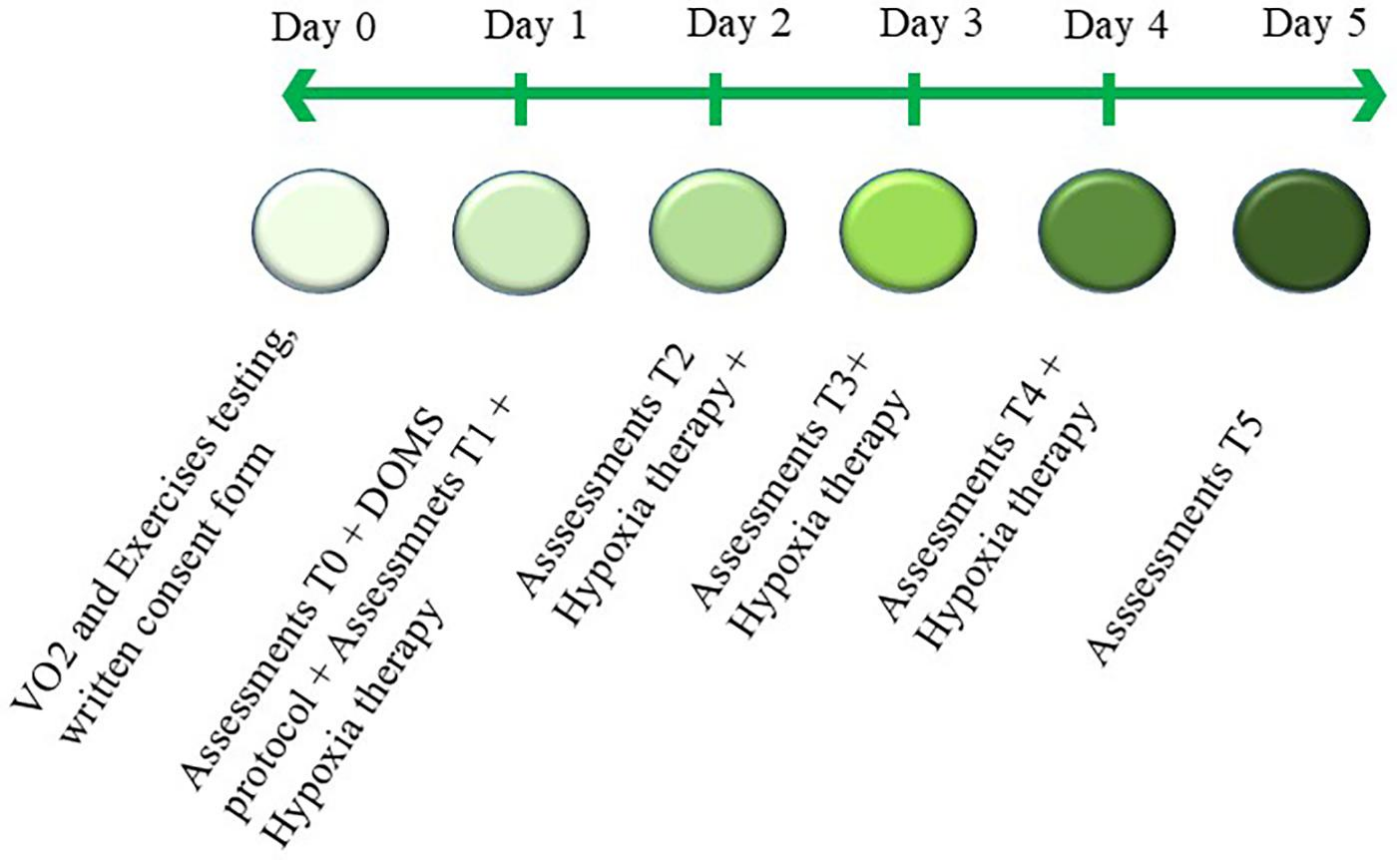
Flow diagram showing the process of the intervention.

The intervention process will last for five days (Figure 1), with breathing intervention administered from Day 1 following the DOMS induction protocol. All participants will be instructed to maintain their habitual training routines.

Additionally, participants will be asked to avoid physical activity for at least 48 h prior to the study, to ensure baseline resting metabolic state.

#### 2.5.1 Day 0: familiarization

During the week prior to DOMS induction and intervention, all participants will attend a familiarization session to become acquainted with the procedures, practice the exercises, undergo an initial graded exercise test to determine VO_2_max, and complete an initial hypoxia trial session. This trial allows the Mitovit device to assess each participant's physiological profile.

#### 2.5.2 Day 1: DOMS induction

Before the DOMS induction, baseline measurements in normobaric conditions will be performed.

The exercise protocol to induce hamstring DOMS through eccentric contractions will include the following exercises: Nordic Hamstring Exercise (5 sets × 5 reps, rest = 90 s); Bilateral Leg Curl (5 sets × 8 reps, 80% 1RM, rest = 90 s); Romanian Deadlift (5 sets × 8 maximal reps, rest = 90 s), and Bilateral Leg Curl eccentric phase only (5 sets × 8 reps, 130% 1RM, rest = 90 s). This protocol was designed based on previous studies (21).

The selected protocol emphasizes high-volume and high-intensity eccentric contractions of the hamstring muscle group, which have consistently been shown to induce exercise-induced muscle damage and delayed onset muscle soreness in physically active individuals (21). Similar eccentric-based protocols have demonstrated reproducible increases in muscle soreness, reductions in strength, and alterations in functional performance within 24–72 h post-exercise (4, 21).

To confirm the success of DOMS induction, subjective measures of muscle soreness and objective neuromuscular performance will be assessed at baseline and daily throughout the intervention period (4). A clinically significant increase in perceived muscle soreness, along with concomitant reductions in strength or performance, will be considered indicative of successful DOMS induction. Creatine kinase (CK), an indicator of muscle damage, will also be assessed to ensure that DOMS has been successfully induced (4).

#### 2.5.3 Days 1–5: intervention and recovery

Following DOMS induction, participants will be assigned to one of three interventions: IH, HH, or control (placebo). Sessions will take place in a controlled environment using a normobaric hypoxia generator. Each session will last 42 min, with participants seated in a reclined position, using the MITOVIT Hypoxic Training System (MITOVIT, Germany). The device automatically adjusts the intensity of the hypoxic or hypoxic–hyperoxic stimulus (FiO_2_) according to individual physiological data collected during the trial session.

##### 2.5.3.1 IH group

Participants will remain at rest and undergo intermittent hypoxia with a 5:2 ratio. Each cycle will consist of 5 min of hypoxia (FiO_2_ ≈ 12%; which corresponds to a simulated altitude of ∼4,300–4,500 m) followed by 2 min of normoxia (FiO_2_ ≈ 21%), totaling six cycles per session. This protocol yields 30 min of hypoxia per session, aiming to achieve an 85% oxygen desaturation threshold. This range safely and effectively activates HIF-1α, triggering cellular adaptations related to angiogenesis, energy metabolism, and mitochondrial biogenesis (22, 23). Each session will last 42 min. IH has been proposed as a non-pharmacological strategy to enhance recovery and physiological adaptations (24).

##### 2.5.3.2 HH group

Participants will remain at rest and undergo hypoxia–hyperoxia with a 5:2 ratio. Each cycle will include 5 min of hypoxia (FiO_2_ ≈ 12%) followed by 2 min of hyperoxia (FiO_2_ ≈ 30%–35%), averaging six cycles per session and totaling 42 min. The protocol aims for a mean desaturation of 85% (25, 26).

The choice of 5-minute hypoxic blocks as the standard duration is based on scientific evidence indicating that this interval is optimal for effectively activating HIF-1α without compromising cellular safety (27).

##### 2.5.3.3 Control group

Participants will remain at rest and will be exposed to continuous normoxia (FiO_2_ ≈ 21%) for 42 min, serving as a reference condition. All participants will remain seated in a relaxed position, breathing through a mask connected to the hypoxia generator. This placebo condition ensures that observed effects are attributable to the active interventions.

#### 2.5.4 Safety considerations

Peripheral oxygen saturation (SpO_2_) and heart rate will be continuously monitored. The system is equipped with two pulse oximeters and prevents SpO_2_ from dropping below the safety threshold of 80%. In addition to real-time SpO_2_ and heart rate monitoring, participants will be verbally screened before each session for symptoms such as dizziness, nausea, headache, excessive fatigue, or discomfort related to mask use (24).

Adverse events will be defined as any unfavorable or unintended signs, symptoms, or physiological responses occurring during or after the intervention sessions, whether or not they are considered related to hypoxic exposure (19). Criteria for immediate interruption of the session will include a sustained SpO_2_ below 80%, abnormal cardiovascular responses, onset of moderate-to-severe symptoms, or participant request to stop the session (24).

In the event of an adverse response, the hypoxic stimulus will be immediately terminated, normoxic breathing will be restored, and the participant will be placed in a seated or supine position as appropriate (24). All adverse events will be documented and reported, and participants experiencing significant adverse responses will be withdrawn from the study and referred for medical evaluation if necessary (19).

Participants will also be advised to drink water after each hypoxia session. Equipment will be disinfected after use with an ozone treatment box (Ozone Treatment and Disinfection Box, JBM, Spain) to eliminate bacteria, viruses, and fungi.

#### 2.5.5 Additional considerations

All participants will be instructed to refrain from engaging in any additional strenuous physical activity involving the lower limbs throughout the intervention period in order to avoid interference with the DOMS recovery process (4). Light daily activities and upper-limb training will be permitted, provided that they do not induce significant muscular fatigue or soreness (4).

### 2.6 Outcomes

Table 1 summarizes the assessment moments of the different outcomes. All outcome assessments will be conducted following a standardized temporal sequence throughout the study period (19). Baseline measurements (Day 0) will be performed prior to DOMS induction and before any hypoxic exposure (19).

**Table 1:** Assessment moments of the different outcomes.

Outcome	Assessment moments
Descriptive variables	Day 0
Physical Recovery variables	Days 1, 2, 3, 4, 5
VO_2_max	Days 0 and 5
Hamstring isometric strength	Days 1, 2, 3, 4, 5
Cognitive function (D2, TMT A/B) and stress (DASS-21)	Days 0 and 5
Psychological profile (CPRD)	Day 0
Sprint 30 m	Days 1, 2, 3, 4, 5
Counter Mouvement Jump (CMJ)	Days 1, 2, 3, 4, 5
Squat 1-RM	Days 1, 2, 3, 4, 5
Inflammatory and recovery biomarquers (HIF-1α, PGC-1α, Lactate, Klotho, CK)	Days 1, 2, 3, 4, 5

On Days 1 to 5, all daily assessments of physical recovery, neuromuscular performance, autonomic nervous system activity, and biochemical markers will be performed at the same time of day for each participant (19). Physical performance outcomes will be collected following a standardized warm-up (28).

The study outcomes will be hierarchically structured as follows: hamstring isometric strength will serve as the primary endpoint; additional physical performance, recovery, and biochemical variables will be treated as secondary endpoints; and cognitive and psychological measures will be analyzed on an exploratory basis.

#### 2.6.1 Descriptive variables

Age, height, and body weight will be measured to calculate body mass index (BMI, kg/m^2^). Physical activity levels (MET-min/week) will be assessed using the International Physical Activity Questionnaire (IPAQ).

#### 2.6.2 Cognitive variables

Given the study's focus on injury prevention, attention and executive function will be assessed using the D2 test and the Trail Making Test (TMT A and B) in Spanish. In these tests, participants are asked to connect numbers and letters in sequential order. Cognition is sensitive to short-term confounding factors such as fatigue induced by the DOMS protocol (18), and we aim to examine whether IH or HH can influence these factors in the short term (29–31). These assessments will be performed on Day 0 (baseline) and Day 5 (post-intervention).

#### 2.6.3 Psychological variables

The Depression, Anxiety, and Stress Scale-21 (DASS-21) will be used, which consists of three self-report scales. Each scale contains 7 items, scored from 0 to 3, where 0 means ‘Not at all’ and 3 means ‘Almost always.’ Scores are summed to obtain a total score for each subscale (32). This variable will be assessed on Day 0 (baseline) and Day 5 (post-intervention).

In addition to these tests, the Psychological Characteristics Related to Sport Performance Questionnaire (CPRD) will be used to assess psychological factors relevant to sports performance. The CPRD measures five dimensions: stress control, influence of performance evaluation, motivation, mental skills, and team cohesion. This instrument has demonstrated robust psychometric properties, indicating high internal consistency (33). This variable will be assessed on Day 0 only, with the intention to perform a recovery sub-group secondary analysis based on personality.

#### 2.6.4 Physical performance variables

Aerobic capacity (VO_2_ max) will be measured on two occasions: day 0 (before the intervention) and day 5 (at the end of the protocol), using the following procedure: First, a warm-up acsm consisting of 5 min of light jogging or walking at 5–6 km/h with a 1% incline will be performed. Next, a ramp protocol will be conducted, starting at 8 km/h with a 1% incline, with speed increased by 0.3 km/h every 30 s while maintaining the incline, until exhaustion or VO_2_ max criteria are reached (34, 35). The test ends when the participant reaches exhaustion, VO_2_ plateau, or 95% of the age-predicted maximum heart rate according to Tanaka's formula [HRmax = 208−0.7 × age]) (36). Finally, a 5-minute cool-down will be performed, walking at 4 km/h with a 0% incline (37). VO_2_ max will be measured using a SpiroFit metabolic analyzer (SpiroFit/Sendsor GmbH, Grafing, Germany), which enables accurate real-time gas exchange assessment during exercise. The SpiroFit operates by measuring real-time gas exchange (O_2_ and CO_2_) during exercise, allowing for precise determination of VO_2_ max by analyzing oxygen consumption and carbon dioxide production, as highlighted by the articles that demonstrate its validity and reliability comparable to standard laboratory methods (38, 39).

The following tests will be conducted on days 1, 2, 3, 4 and 5:

**30-m sprint speed:** Sprint will be assessed using Witty photocells (Microgate, Bolzano, Italy), which measures velocity over a 30-meter sprint. This test will help evaluate speed and acceleration, key components of athletic performance. A three-minute rest period will be applied between the two attempts to allow full recovery (40).
**Countermovement jump (CMJ):** The CMJ is a standing exercise in which the hands are placed on the hips (41). The movement follows the typical eccentric-isometric-concentric sequence of plyometric training, consisting of a downward knee flexion followed by an explosive upward jump. This test will be measured using the MyJump2 app (42), a mobile application for video-based analysis of vertical jump performance used by coaches, athletes, and researchers to assess jump metrics without laboratory equipment. It utilises the smartphone camera to time jump events and derive kinetic and kinematic performance parameters. Each participant will have two attempts, with a three-minute rest period in a standing position between jumps to allow their energy systems to stabilize (43).
**Maximum strength (1-RM):** Maximal dynamic strength in the half-squat will be assessed using a Smith machine to prevent anteroposterior oscillations that could interfere with execution velocity measurements (44). A Vitruve VBT encoder (Vitrube, Spain) will be firmly attached to one end of the bar to avoid interfering with the movement. This encoder measures the vertical displacement velocity of the bar (meters per second) and allows 1-RM estimation using the equations by González-Badillo et al. (45). The exercise will be performed with a load equivalent to 80% of each participant's body weight (46). Participants will have two attempts to complete the test, with the best result recorded each time. A three-minute rest interval will be applied between attempts to ensure adequate recovery (47).
**Isometric strength:** Maximal voluntary isometric strength will be assessed using an activForce 2 dynamometer. Participants will be asked to generate maximal force in a static position. Since hamstring activity is being recorded, the participant will sit with the knee at a 90-degree angle and the ankle properly positioned on the dynamometer pad, ensuring that the force is transmitted through the hamstrings muscle group while maintaining proper posture and stability. The highest force produced during the trial will be recorded (48).

#### 2.6.5 Physical recovery variables

All physical recovery variables will be measured daily on days 1, 2, 3, 4, and 5, before the hypoxia intervention to ensure participants are in a non-fatigued and fully recovered state.

Pressure Pain Threshold (PPT): A mechanical pain threshold analysis will be performed using a Baseline algometer (Wagner instruments, Greenwich, United States). Measurements will be taken on the midpoint of the dominant leg's hamstring muscle belly, defined as the midpoint between the ischial tuberosity and the popliteal crease. Participants will use the Visual Analog Scale (VAS) to rate their hamstring pain on a scale from 0 to 10, where 0 represents no pain and 10 represents the worst possible pain. To ensure reproducibility, three measurements will be taken at each site with a 30-second interval between trials, and the mean value will be used for analysis. The same examiner, trained in algometry procedures, will perform all measurements (49, 50).

Muscle oxygen saturation (SmO_2_) will be monitored in real time with a Moxy Monitor (Fortiori Design LLC, Hutchinson, MN, USA) placed on the hamstrings muscle group, while the participant is performing an effort during the 30 m sprint (51).

Autonomic nervous system (ANS) activity will be assessed using heart rate variability (HRV) measured with a Polar H10 chest strap (Polar Electro Oy, Kempele, Finland). The strap will be positioned around the participant's thorax, just below the sternum and in contact with the skin at the level of the xiphoid process, ensuring firm placement for optimal signal acquisition. Measurements will be taken in a seated, rested position for 5 min, and participants will be instructed to avoid speaking or moving during the recording. To ensure reproducibility, each measurement will be performed at the same time of day, by the same trained investigator, and the mean of three consecutive recordings will be used for analysis. Polar H10 chest straps have been shown to provide high validity and reliability for HRV measurement compared to standard ECG systems in both laboratory and field settings (52). Beyond standard HRV metrics, advanced ANS indices will be extracted using validated signal-processing algorithms to characterize sympathetic and parasympathetic modulation more comprehensively. Variables such as the Stress Index, parasympathetic activation scores, and overall autonomic balance will be collected each day before the hypoxia intervention (53). These indicators will allow assessment of physiological stress, recovery status, and autonomic regulation throughout the protocol, offering an additional layer of interpretation regarding the systemic impact of DOMS combined with intermittent hypoxia (53).

Active knee extension range of motion: Participants will be positioned supine with the hip joint maintained at 90° of flexion. They will be then instructed to voluntarily extend the knee as far as possible while keeping the hip flexion locked at 90°. In this phase, hamstring flexibility will be assessed by measuring the knee extension range of motion with the App *Goniometer Pro* (Digiflex Labs/Avvi LLC, iOS) (54).

Body composition will be assessed using the BIODY XPERT ZM3 (Aminogram SAS/eBIODY, La Ciotat, France) device, which provides multi-frequency bioelectrical impedance measurements, including the phase angle (55). Measurements will be performed under standardized conditions each day before the hypoxia intervention to ensure consistency. The phase angle, derived from the relationship between reactance and resistance, reflects cellular integrity and membrane functionality. Although it does not directly quantify oxidative stress, reductions in phase angle have been associated with increased cellular stress, altered membrane permeability, and elevated inflammatory or oxidative processes (56). Thus, in this study, the phase angle will be used as an indirect biomarker of cellular stress potentially influenced by intermittent hypoxia and DOMS (56, 57). Daily monitoring will allow evaluation of acute fluctuations in cell health and their relationship with performance and recovery markers.

#### 2.6.6 Biochemical markers

The selection of biochemical markers in this study was designed to capture complementary aspects of the physiological response to exercise-induced muscle damage and hypoxic-based recovery interventions, rather than to provide redundant information (13, 58). Specifically, the chosen biomarkers reflect molecular signaling, metabolic stress, muscle damage, and systemic recovery processes relevant to DOMS (4).

Inflammatory markers: This procedure will be repeated on days 1, 2, 3, 4, 5. On day 1, the first measure will be taken prior to any physical activity, and the second will be taken right before the hypoxic intervention. On days 2, 3, 4, 5 there will only be one measurement prior to the hypoxic intervention. This sampling schedule is designed to capture the systemic inflammatory response that emerges approximately 24 h after exercise-induced muscle damage, rather than the acute changes that occur immediately post-exercise, which are more transient and variable. Measuring prior to the hypoxic intervention on subsequent days allows assessment of the 24-h post-stimulus inflammatory status, reflecting the cumulative effects of prior activity and recovery processes (59). Various biomarkers related to inflammation, muscle damage, and stress response will be assessed, including HIF-1α, PGC-1α, and Klotho protein.

To assess inflammatory markers, venous blood samples (∼5 mL per sample) will be collected by a trained nurse via venipuncture using EDTA-containing tubes to prevent coagulation. The venipuncture procedure will be carried out as follows: the participant will sit in a chair, and the nurse will locate an accessible vein in the arm. Once the vein is identified, the area will be disinfected, and a sterile single-use needle will be inserted to draw the blood sample in vacutainer tubes. Prior to sampling, the site will be cleaned with an alcohol swab, and standard aseptic procedures will be followed to minimize contamination and hemolysis. After collection, samples will be immediately placed on ice and processed within 1 h, including centrifugation to separate serum or plasma, and stored at −80 °C until analysis. Subsequently, plasma will be separated by centrifugation at 2000–2500×*g* for 15 min at 4 °C and rapidly stored at –80 °C to preserve biomarker integrity. Concentrations of specific biomarkers will be quantified using enzyme-linked immunosorbent assay (ELISA) kits (R&D Systems, Minneapolis, MN, USA) according to the manufacturer's protocol. For HIF-1α, in addition to ELISA, Western Blot will be employed as a complementary technique to confirm protein expression and better evaluate its regulation in response to hypoxia (60).

HIF-1α is a key transcription factor in the hypoxic response. Its activation regulates the expression of genes involved in cellular adaptation to low-oxygen conditions, such as energy metabolism and angiogenesis. HIF-1α was selected as a primary molecular marker of hypoxic signaling and cellular adaptation to reduced oxygen availability (13).

PGC-1α is a transcriptional coactivator involved in mitochondrial biogenesis and exercise adaptation. Its expression increases following physical activity, promoting muscular endurance and metabolic efficiency. Assessing its levels will provide information on cellular adaptation capacity and recovery potential (58). PGC-1α will be assessed using complementary molecular techniques. Quantitative PCR will be employed to measure mRNA expression levels, while Western blot analysis will be conducted to confirm protein abundance. For qPCR, total RNA will be extracted from peripheral blood mononuclear cells obtained from venous blood samples collected from participants. RNA isolation will be performed using commercially available RNA extraction kits following the manufacturer's general instructions. This dual approach ensures comprehensive evaluation of both transcriptional and translational regulation of PGC-1α.

Klotho is a protein associated with healthy aging and cellular protection against oxidative stress, inflammation, and tissue damage. Its expression has been linked to improved muscle regeneration, mitochondrial function, and insulin sensitivity, key aspects during post-exercise recovery. Following intermittent hypoxia or hypoxia-hyperoxia interventions, Klotho levels are expected to increase as part of the body's adaptive response. Analyzing this protein will allow evaluation of the systemic impact of recovery strategies on redox balance, cellular longevity, and metabolic performance (61).

Additionally, CK and blood lactate were selected as established markers of muscle damage and metabolic stress, respectively, allowing confirmation of exercise-induced muscle stress and characterization of recovery dynamics across the intervention period (4, 62). Blood lactate and creatine kinase (CK) levels will be measured using capillary blood samples obtained via safety lancets from the fingertip. All samples will be collected following standard aseptic procedures to ensure accuracy and minimize discomfort.

Blood lactate concentration will be measured using a portable analyzer (Lactate Scout Sport, EKF Diagnostics, Cardiff, UK). Lactate levels are expected to be elevated after demanding exercises, particularly those involving eccentric contractions, and will be used as an important marker to detect metabolic stress, fatigue, and to monitor physiological adaptation following intense physical activity (62, 63).

CK levels will be measured as a key biomarker to assess muscle damage and recovery following intense physical activity involving eccentric contractions. For the analysis, the SimplexTAS 101 point-of-care clinical chemistry and immunoassay analyzer (TASCOM Co., Ltd., Anyang-si, Gyeonggi-do, Republic of Korea) will be used.

### 2.7 Statistical analysis

All statistical analyses will be performed using IBM SPSS Statistics version 29 (IBM Corp., Armonk, NY, USA). The primary confirmatory analysis will focus on hamstring isometric strength. The normality of the variables will be assessed using the Shapiro–Wilk test. Descriptive analysis will be performed using the mean ± standard deviation for normally distributed data, or the median and interquartile range for data not following a normal distribution.

The principal analysis will evaluate the Group × Time interaction using repeated-measures ANOVA, as this term reflects the differential effect of the intervention over time between groups. When significant interaction or main effects are observed, Bonferroni-adjusted *post hoc* comparisons will be conducted. Effect size will be evaluated using partial eta squared (*η*^2^*p*).

Analyses will be conducted according to the intention-to-treat principle, including all randomized participants in the groups to which they were allocated. Missing data will be handled using multiple imputation procedures under the missing-at-random assumption prior to conducting the repeated-measures ANOVA.

Secondary outcomes will be analyzed using the same analytical approach. Where multiple comparisons across secondary endpoints are conducted, Bonferroni correction will be applied within each family of related outcomes.

Cognitive and psychological variables will be analyzed on an exploratory basis. Correlation analyses (Pearson or Spearman) and multiple linear regression models will be performed to explore potential associations and predictors of cognitive improvement or functional recovery. The level of statistical significance will be set at *p* < 0.05.

## 3 Dissemination plan

The results of this study will be disseminated through multiple channels to ensure broad visibility among researchers, clinicians, and practitioners in sports science and rehabilitation. Primary dissemination will occur via publication in a peer-reviewed open-access journal, ensuring unrestricted access to the scientific community. Findings will also be presented at national and international conferences related to sports medicine, exercise physiology, and hypoxic conditioning.

In addition, summaries of the study outcomes will be shared with participating institutions, sports professionals, and relevant stakeholders through professional networks and academic platforms. Where appropriate, results may be translated into practical recommendations and disseminated via workshops, webinars, or continuing education activities targeting coaches, physiotherapists, and sports clinicians. This multi-level dissemination strategy aims to facilitate both scientific impact and real-world application of hypoxia-based recovery interventions.

## 4 Discussion

DOMS remains a common consequence of unaccustomed or high-intensity eccentric exercise, and current recovery strategies show variable and often inconsistent efficacy (4). Consequently, there is a growing need for innovative, non-pharmacological interventions that can enhance muscle recovery while minimizing adverse effects. IH and HH have demonstrated beneficial physiological adaptations in other contexts, including improved oxygen utilization, vascular function, and systemic stress tolerance, yet their role in DOMS recovery has not been systematically investigated (6–8).

This study protocol describes the first randomized controlled trial designed to evaluate the effects of IH and HH on recovery from exercise-induced muscle damage. A major strength of the proposed design is its integrative approach, combining physiological, biochemical, cognitive, and psychological outcomes. This comprehensive assessment will allow for a more nuanced understanding of how hypoxic-based interventions may influence not only muscle recovery but also mental performance and overall adaptation to physical stress.

The findings of this trial have the potential to inform future research on hypoxic conditioning and to support the development of evidence-based recovery strategies for athletes and physically active individuals. If proven effective, IH and HH could represent practical and scalable alternatives to traditional recovery methods, with implications for sports performance, rehabilitation, and clinical practice.
